# The BODE (Body Mass Index, Airflow Obstruction, Dyspnea, and Exercise Capacity) Index in Chronic Obstructive Pulmonary Disease: A Comprehensive Clinical Assessment Tool

**DOI:** 10.7759/cureus.95206

**Published:** 2025-10-23

**Authors:** Mashal Mumtaz, Sharmila Venkatachalapathi, Taher Mohammed, Parikibanda Sudeshna, Tulasi Kota, Stefy S Gandhi, Mariam Melkumian, Zaki Ur Rahman Mohammed, Kiranjot Kaur, Riyan Khalid

**Affiliations:** 1 Internal Medicine, University College of Medicine and Dentistry, University of Lahore, Lahore, PAK; 2 Internal Medicine, Periyar Government Hospital, Mayiladuthurai, IND; 3 Internal Medicine, Vadamalayan Hospitals Pvt Ltd., Dindigul, IND; 4 Medical Education, StepExcel Boards Academy, Skokie, USA; 5 Medicine, StepExcel Boards Academy, Skokie, USA; 6 Internal Medicine, Valparaiso University, Valparaiso, USA; 7 Medicine, D Y Patil University, School of Medicine, Navi Mumbai, IND; 8 Research, StepExcel Boards Academy, Skokie, USA; 9 Internal Medicine, University of North Dakota, Grand Forks, USA; 10 Internal Medicine, United States Navy, United States Military, North Chicago, USA; 11 Clinical Research, Arizona State University, Tempe, USA; 12 Internal Medicine, Shri B M Patil Medical College, Bijapur, IND; 13 Internal Medicine, Jinnah Postgraduate Medical Centre, Karachi, PAK

**Keywords:** bode index, copd, exacerbations, mortality, multidimensional assessment, pulmonary rehabilitation

## Abstract

Chronic obstructive pulmonary disease (COPD) is a heterogeneous condition with systemic effects extending beyond airflow limitation. Spirometry alone is insufficient for a comprehensive assessment. The BODE index, which integrates body mass index, airflow obstruction, dyspnea, and exercise capacity, was developed to improve prognostication by capturing multiple dimensions of disease burden.

This systematic review, conducted in accordance with Preferred Reporting Items for Systematic Reviews and Meta-Analyses (PRISMA) 2020 guidelines, searched PubMed, Embase, Scopus, and the Cochrane Library up to August 2025. Eligibility was defined using the PICO framework, and risk of bias was assessed with the Newcastle-Ottawa Scale and QUIPS tool.

From 128 records, five studies comprising 2,482 patients with COPD were included. The BODE index consistently outperformed FEV₁ and GOLD staging in predicting mortality, exacerbations, and hospitalizations. Modified indices, such as the ADO (age, dyspnea, obstruction) and i-BODE (incremental shuttle walk test in place of six-minute walk distance), enhanced feasibility and calibration in primary care and rehabilitation settings. Across studies, outcome ascertainment was robust, with an overall low to moderate risk of bias.

The BODE index provides a multidimensional and clinically meaningful approach to COPD evaluation, surpassing spirometry by reflecting both pulmonary and systemic disease burden. It supports better risk stratification for clinical management and research. Future studies should explore integration with biomarkers, imaging, and digital tools to refine prognostic accuracy and guide personalized care.

## Introduction and background

Chronic obstructive pulmonary disease (COPD) is a progressive respiratory condition characterized by persistent airflow limitation and an abnormal inflammatory response of the lungs to noxious particles or gases. It affects millions of individuals worldwide and is among the top three leading causes of death globally [1]. Beyond its high mortality, COPD is associated with recurrent exacerbations, reduced quality of life, and substantial healthcare utilization, making it a significant socioeconomic burden [2]. Traditionally, the severity of COPD has been assessed through spirometry, particularly the measurement of forced expiratory volume in one second (FEV₁).

This parameter has long served as the basis of the Global Initiative for Chronic Obstructive Lung Disease (GOLD) staging system [3]. However, FEV₁ alone provides a limited view of disease status. Clinical observations and epidemiological data consistently demonstrate that patients with similar levels of airflow limitation may present with very different symptoms, exercise capacities, nutritional profiles, and survival outcomes [4]. This variability emphasized the need for a more comprehensive approach to disease assessment. COPD is not solely a pulmonary disorder; it also involves systemic consequences such as weight loss, skeletal muscle dysfunction, reduced exercise tolerance, and psychological distress. These systemic manifestations influence prognosis as much as, or sometimes more than, airflow limitation. To address this complexity, multidimensional indices were developed to capture the broader clinical spectrum of COPD.

Among these tools, the BODE index, which is an acronym for Body Mass Index (B), Airflow Obstruction (O, measured by FEV₁), Dyspnea (D, assessed by the Medical Research Council (MRC) dyspnea scale), and Exercise capacity (E, measured by the six-minute walk distance (6MWD)), has become one of the most widely validated prognostic indices [5]. By integrating nutritional status, lung function, perceived breathlessness, and functional performance, the BODE index reflects the interaction between pulmonary impairment, systemic involvement, and patient-centered outcomes. The BODE scoring system stratifies patients into risk categories that correlate with survival, risk of exacerbations, and healthcare utilization. This multidimensional approach provides clinicians with a comprehensive framework for prognostication and management. Importantly, it highlights COPD as a systemic disorder rather than a condition confined only to the lungs.

The clinical significance of multidimensional indices such as the BODE index extends beyond mortality prediction. They can guide treatment decisions, optimize healthcare resource allocation, and identify high-risk patients who may benefit from targeted interventions such as pulmonary rehabilitation, intensive follow-up, or preventive therapies [6]. With the rising emphasis on personalized medicine, these indices serve as a bridge between physiological measurements and real-world patient outcomes. This systematic review therefore examines the role of the BODE index in COPD, with a focus on its clinical utility, physiological basis, prognostic implications, and modifications designed to improve feasibility and applicability across diverse healthcare settings.

## Review

Materials and methods

Search Strategy

A systematic search of the literature was conducted across PubMed, Embase, Scopus, and the Cochrane Library from inception until August 2025. Keywords and MeSH terms included “Chronic Obstructive Pulmonary Disease,” “COPD,” “BODE Index,” “Body mass index, airflow obstruction, dyspnea, exercise capacity,” “prognosis,” “mortality,” and “hospitalization.” Boolean operators were used to combine terms appropriately. Reference lists of relevant publications were screened manually to capture additional studies. The search process was designed to be comprehensive, ensuring coverage of both original articles and validation studies. Although no restrictions were applied during the search, only full-text English-language articles were included for final analysis. The Preferred Reporting Items for Systematic Reviews and Meta-Analyses (PRISMA) 2020 guidelines were followed throughout the review process [7].

Eligibility Criteria

Eligibility was determined using the PICO framework [8]. The Population included adults aged 40 years or older with a confirmed diagnosis of COPD based on GOLD or equivalent criteria. The Intervention was the use of the BODE index or its validated modifications such as the ADO or i-BODE. The Comparator was conventional measures of severity, including FEV₁ or GOLD staging, and other prognostic indices. The Outcomes included mortality, frequency of exacerbations, hospitalization risk, and predictive accuracy. Exclusion criteria included case reports, reviews, editorials, animal studies, and conference abstracts. Studies not reporting BODE-related outcomes were also excluded.

Study Selection

Following the initial search, all records were imported into a reference management system, and duplicates were removed. Two independent reviewers screened the titles and abstracts for relevance. Articles deemed suitable were then assessed in full text to confirm eligibility. Studies were excluded if they did not meet the PICO criteria, lacked outcome data, or represented duplicate datasets. Any discrepancies between reviewers were resolved through consensus discussions. The screening process ensured that only high-quality studies addressing the prognostic role of the BODE index were retained.

Data Extraction

A standardized form was used to extract data from each study. Key variables included author, year, country, study design, sample size, and patient population. Details of the intervention (BODE index or modified versions), comparators (such as FEV₁ or GOLD stage), and primary outcomes were recorded systematically. Physiological rationale, findings, and clinical relevance were also documented. Data extraction was performed independently by two reviewers to minimize error. Consensus was achieved through joint verification of the extracted tables and narrative summaries.

Risk of Bias Assessment

Risk of bias was independently assessed by two reviewers. Cohort studies were evaluated using the Newcastle-Ottawa Scale (NOS), focusing on participant selection, comparability, and outcome assessment [9]. Prognostic validation studies were assessed with the Quality in Prognostic Studies (QUIPS) tool [10]. Each study was rated as low, moderate, or high risk of bias. Disagreements were resolved by consensus. The assessment process allowed for a critical appraisal of study strengths and limitations, ensuring balanced interpretation of findings. These results were incorporated into evidence synthesis and tables summarizing the methodological quality.

Data Synthesis

Due to heterogeneity in study design, populations, and outcome reporting, a meta-analysis was not performed. Instead, a narrative synthesis was undertaken, highlighting mortality, exacerbations, hospitalization risk, and index modifications. Results were organized into structured tables for easier interpretation. Each study’s contribution was analyzed within the broader context of COPD management and prognostication. Physiological underpinnings of each index were also considered to explain observed associations. This approach allowed for a comprehensive evaluation while acknowledging variability in methods and populations.

Ethical Considerations

This review analyzed data from previously published studies and did not involve direct human participation; therefore, ethical approval was not required. The review was conducted in accordance with the Preferred Reporting Items for Systematic Reviews and Meta-Analyses (PRISMA) 2020 guidelines. Although the protocol was not registered in the International Prospective Register of Systematic Reviews (PROSPERO), all steps were followed transparently to ensure reproducibility and methodological rigor.

Results

Study Selection Process

**Figure 1:**
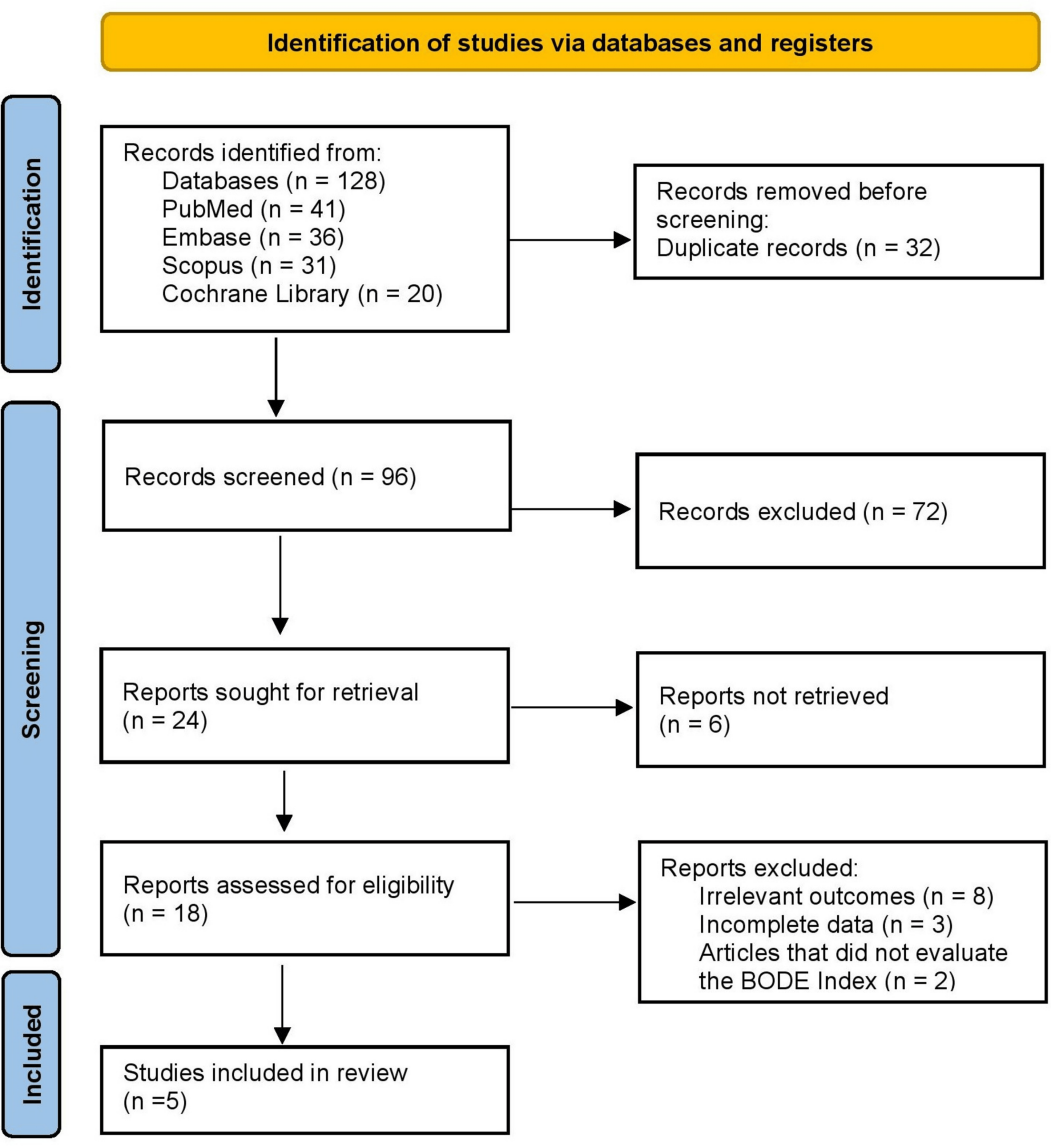
PRISMA 2020 Flow Diagram PRISMA: Preferred Reporting Items for Systematic Reviews and Meta-Analyses; BODE: Body Mass Index, Airflow Obstruction, Dyspnea, and Exercise Capacity

Figure 1 shows database search initially identified 128 records, of which 41 were from PubMed, 36 from Embase, 31 from Scopus, and 20 from the Cochrane Library. Following removal of 32 duplicate records, a total of 96 unique studies were screened by title and abstract. At this stage, 72 records were excluded for not being relevant to the research question. The remaining 24 full-text reports were sought for retrieval, and six articles were not successfully retrieved. After detailed eligibility assessment of 18 studies, 13 studies were excluded, including eight with irrelevant outcomes and three with incomplete data, along with two articles that did not evaluate the BODE index. Ultimately, five studies met the inclusion criteria and were included in this systematic review.

Characteristics of the Selected Studies

Table 1 summarizes the five included studies on the prognostic role of the BODE index in COPD. Celli et al. validated the index in 832 patients, showing it predicted all-cause and respiratory mortality better than FEV₁ or GOLD staging [11]. Marin et al. studied 275 outpatients and found that higher BODE quartiles correlated with more frequent and severe exacerbations [12]. Ong et al. confirmed in 127 patients that the BODE index outperformed FEV₁ and GOLD in predicting hospitalizations and mortality, linking exercise tolerance to systemic frailty [13]. Puhan et al. evaluated 574 patients across Swiss and Spanish cohorts and demonstrated that updated BODE and ADO indices improved calibration for three-year mortality and generalizability in primary care [14]. Moberg et al., in 674 patients undergoing pulmonary rehabilitation, validated the i-BODE index, showing that substituting the incremental shuttle walk test (ISWT) for the 6MWD preserved prognostic accuracy and enhanced feasibility [15]. Collectively, these studies highlight the BODE index and its modifications as reliable multidimensional tools for mortality, exacerbation, and hospitalization risk assessment in COPD.

**Table 1:** Characteristics of the Selected Studies BMI: Body Mass Index; FEV₁: Forced Expiratory Volume in 1 second; GOLD: Global Initiative for Chronic Obstructive Lung Disease; 6MWD: Six-Minute Walk Distance; ISWT: Incremental Shuttle Walk Test; ADO: Age, Dyspnea, Obstruction Index; i-BODE: Incremental Shuttle Walk-Based BODE Index; BODE: Body Mass Index, Airflow Obstruction, Dyspnea, and Exercise Capacity

Authors & Year	Population (P)	Exposure/Condition (I)	Comparator (C)	Outcomes (O)	Physiology	Findings	Importance
Celli et al., 2004 [11]	832 COPD patients (derivation: 207; validation:625)	BODE Index (BMI, FEV₁, dyspnea, 6MWD)	FEV₁ alone; GOLD staging	All-cause and respiratory mortality	Captures systemic physiology: ventilatory limitation, exercise tolerance, nutritional status	Higher BODE quartiles strongly predicted mortality	Landmark study; validated multidimensional tool
Marin et al., 2009 [12]	275 COPD outpatients	BODE quartiles	FEV₁	Exacerbations (frequency, severity, time to first)	Reflects inflammation-linked exacerbator phenotype	Higher BODE = more frequent/severe exacerbations	Extended utility beyond mortality
Ong et al., 2005 [13]	127 COPD patients	BODE quartiles	GOLD, FEV₁	Hospitalizations, mortality	Physiological link: exercise tolerance mirrors systemic frailty	BODE predicted hospitalization risk better than FEV₁	Reinforced healthcare utilization link
Puhan et al., 2009 [14]	574 COPD (Swiss: 232, Spanish: 342)	Updated BODE & ADO indices	Original BODE	3-year mortality; calibration accuracy	Pathophysiology embedded via dyspnea, obstruction, age	Better calibration; ADO practical in primary care	Improved generalizability; external validation
Moberg et al., 2014 [15]	674 COPD patients in pulmonary rehab	i-BODE (ISWT instead of 6MWD)	Original BODE	Mortality, time to hospitalization	ISWT reflects exercise limitation; comparable physiology to 6MWD	ISWT substitution maintained prognostic power	Enhances feasibility in rehab programs

Risk of Bias Assessment

Table 2 presents the risk of bias assessment for the included studies. Celli et al. studied a prospective cohort with derivation and validation groups, assessed using the NOS, and rated low-moderate due to strong design but minor selection bias from specialty clinics [11]. Marin et al. studied a multicenter prospective cohort, which was evaluated with NOS and QUIPS, showing low risk because of prespecified outcomes, strong follow-up, low attrition, and adjusted analyses [12]. Ong et al. assessed a single-center historical cohort, which was rated moderate risk on NOS given its small sample and limited generalizability, though hospitalization outcomes were reliable [13]. Puhan et al. studied multicenter calibration and validation cohorts, which scored low risk with NOS and QUIPS due to independent validation, rigorous calibration, and confounder adjustment [14]. Finally, Moberg et al. assessed a prospective rehabilitation cohort, which was rated moderate risk under NOS and QUIPS since external validity was limited to rehab populations, though mortality ascertainment was robust through registries [15].

**Table 2:** Risk of Bias Assessment NOS: Newcastle-Ottawa Scale; COPD: Chronic Obstructive Pulmonary Disease; QUIPS: Quality in Prognostic Studies Tool; BODE: Body Mass Index, Airflow Obstruction, Dyspnea, and Exercise Capacity

Authors & Year	Study Design	Risk of Bias Tool	Risk of Bias Rating	Justification
Celli et al., 2004 [11]	Prospective cohort (derivation + validation)	Newcastle–Ottawa Scale (NOS)	Low–Moderate	Large, well-designed cohorts; robust outcomes; minor selection bias from specialty clinics.
Marin et al., 2009 [12]	Multicenter prospective cohort	NOS/QUIPS	Low	Prespecified outcomes (exacerbations); strong follow-up; low attrition; adjusted analyses.
Ong et al., 2005 [13]	Single-center historical cohort	NOS/QUIPS	Moderate	Small sample size; single-center; hospitalization data reliable but generalizability limited.
Puhan et al., 2009 [14]	Multicenter calibration/validation cohorts	NOS/QUIPS	Low	Independent validation cohorts; rigorous calibration; comprehensive adjustment for confounders.
Moberg et al., 2014 [15]	Prospective pulmonary rehab cohort	QUIPS	Moderate	Selected rehab population limits external validity; mortality ascertainment strong

Discussion

The BODE index has become one of the most important multidimensional tools for assessing prognosis in COPD because it integrates variables that represent different facets of the disease. Each component reflects a unique pathophysiological process that influences long-term outcomes. Low BMI is a marker of systemic wasting and muscle dysfunction, which are common in advanced COPD and strongly associated with higher mortality [16]. Airflow obstruction, captured by FEV₁, reflects structural and inflammatory lung damage that limits ventilation. Dyspnea, quantified using the MRC scale, represents the symptomatic burden of disease and correlates with functional status and patient-reported quality of life. Finally, exercise capacity, measured by the 6MWD, serves as an integrated marker of cardiopulmonary reserve, skeletal muscle function, and overall frailty [17]. By combining these domains, the BODE index synthesizes structural, functional, systemic, and subjective aspects of COPD into a single clinically meaningful score.

The prognostic value of the BODE index has been demonstrated consistently. Celli et al. (2004) showed that each one-point increase in the index was associated with a measurable rise in mortality risk, while higher quartiles were linked to shorter survival [11]. Subsequent studies expanded this evidence: Marin et al. (2009) reported that higher BODE quartiles correlated with more frequent exacerbations and earlier recurrence [12]. Ong et al. (2005) reinforced these findings, showing that higher scores predicted hospitalizations and identified patients likely to consume more healthcare resources [13]. Because exacerbations and hospitalizations accelerate disease progression, worsen quality of life, and increase costs, these results confirm the BODE index as a powerful prognostic tool. Over time, adaptations have aimed to improve feasibility while preserving accuracy. Puhan et al. (2009) introduced the ADO Index (Age, Dyspnea, Obstruction), which replaced exercise testing with age, improving calibration in primary care and among older adults for whom walk tests may not be practical [14]. Similarly, Moberg et al. (2014) validated the i-BODE index, substituting the ISWT for 6MWD, and showed that it maintained prognostic accuracy in rehabilitation cohorts [15]. These adaptations underscore the flexibility of the BODE framework across diverse healthcare settings.

Beyond these modifications, comparisons with other multidimensional indices provide additional perspective. The DOSE (Dyspnea, Obstruction, Smoking, Exacerbation) index, developed by Jones et al. (2009), is simpler to apply in clinical practice because it does not require exercise testing, and it predicts exacerbations and hospitalizations effectively [18]. However, its predictive power for long-term mortality is less robust than BODE. The COTE (COPD-specific Comorbidity Test) index, introduced by Divo et al. (2012), incorporates 12 weighted comorbidities that strongly influence survival [19]. Unlike BODE, which emphasizes pulmonary and functional domains, COTE highlights the systemic burden of comorbid conditions. Importantly, combining COTE with BODE has been shown to enhance prognostic accuracy for mortality compared with either score alone. These findings suggest that while BODE remains the most widely validated multidimensional index, DOSE and COTE offer complementary insights that may be particularly useful in routine practice and comorbidity management.

Despite its strengths, some limitations must be acknowledged. Several studies, including those by Marin et al. (2009) and Ong et al. (2005), had relatively small sample sizes, which may reduce precision and generalizability [12,13]. Heterogeneity in exercise testing methods (6MWD vs. ISWT) complicates direct comparisons across studies. Moreover, most cohorts were drawn from outpatient or rehabilitation settings, which may not represent broader community COPD populations. Finally, while the BODE index is firmly established as a prognostic tool, its role in guiding treatment decisions or monitoring response to therapy remains to be fully defined.

Future studies should directly compare the BODE index with other multidimensional tools such as DOSE and COTE across diverse clinical populations to determine their relative strengths. Integration of the BODE framework with biomarkers, imaging modalities, and digital health platforms may further refine its prognostic accuracy. Large-scale, community-based studies are needed to validate its generalizability beyond specialty cohorts. Finally, exploring its role in guiding therapeutic decisions and monitoring treatment response could transform the BODE index from a prognostic marker into a practical tool for personalized COPD management.

## Conclusions

The BODE index offers a multidimensional approach to COPD assessment by combining nutritional status, airflow obstruction, dyspnea, and exercise capacity into a single score. It surpasses spirometry alone by predicting not only survival but also exacerbations and hospitalizations, thereby capturing both pulmonary and systemic disease burden. In clinical practice, it enables more precise risk stratification and tailored interventions, while in research it provides a validated tool for patient grouping and outcome evaluation. Although variability in populations and methods exists, the BODE index remains central to COPD prognostication. Future integration with biomarkers, comorbidity indices such as COTE, and digital health tools has the potential to further enhance its role in delivering personalized care.
